# Supplementary material for: Are carbon emissions trading and green financial instruments synergistic? -Comprehensive quantitative research based on content analysis
Source: PLoS One. 2024 Mar 7;19(3):e0298601. doi: 10.1371/journal.pone.0298601 (PMC10919601; doi:10.1371/journal.pone.0298601)
Supplement: S1 Data — (ZIP) [file pone.0298601.s001.zip › Data/地级市Z_F工作报告.zip!/║ú─╧/╚2╤╟╩╨/└┤╫╘╣1⁄2╓┌║┼í╛┬φ┐╦╩2╛▌═°í┐_2017.docx]

各位代表:

现在,我代表市政府向大会报告工作,请予审议,并请市政协委员和其他列席人员提出意见。

一、2016年工作回顾

过去一年,在省委、省政府和市委的正确领导下,在市人大、市政协的监督支持下,市政府坚持稳中求进,改革创新,适应发展新常态,践行发展新理念,经济社会稳定健康发展,城市面貌发生巨大变化,实现了“十三五”良好开局。预计全市实现生产总值478亿元(含农垦,未经省统计局评估),增长7.5%;固定资产投资782亿元,增长10.8%;社会消费品零售总额196亿元,增长8%;地方一般公共预算收入89.6亿元,增长8.2%;城镇、农村常住居民人均可支配收入分别为31113元和13365元,分别增长8.1%和9.3%。我们过去一年主要抓好了以下工作:

(一)抓项目扩投资,经济保持稳定发展。从落实项目用地、提高审批效率、加强属地管理、抓好督查督办等方面入手,多措并举推进项目建设。2016年市重点项目完成投资540亿元,增长17%,“服务社会投资百日大行动”计划新开工项目已全部开工。一批基础设施、重点产业、城市“双修”、民生保障等领域的重大项目加快推进。崖州中心渔港正式启用,海棠湾红树林度假酒店正式开业,今年春节前,三环路凤凰机场段、海榆西线凤凰路至解放四路段将实现通车,水稻公园小镇一期工程将投入运营;凤凰机场、凤凰岛国际邮轮港加快扩建,凤凰机场综合楼已启用,海棠湾亚特兰蒂斯、梦幻不夜城等项目加快推进;三亚新机场、有轨电车T1线、南海佛学院等项目开工建设,中船重工、中船工业、人大附中、北大口腔医院、格莱美音乐文化中心等著名机构和优质项目落户三亚。

(二)抓产业调结构,转型升级逐步加快。2016年全市三次产业增加值比例为14.4:19.7:65.9。旅游业提质增效,邮轮游艇、低空旅游等业态加快发展,内河观光游开始运营,旅游铁路、海上巴士春节前将开通运营,华侨城、长隆集团引进事宜有序开展,16个美丽乡村示范村启动建设。获评全国旅游标准化示范城市,实行“四位一体”旅游监管机制,12345、12301热线并线运行,及时处理投诉,开展明察暗访,坚决严管重罚,提升了游客满意度。2016年全市接待过夜游客1647万人次,实现旅游总收入354亿元,分别增长10.1%和17%;入境游客止跌回升,达到43.6万人次,增长22%。医疗健康产业实现新突破,恒大国际合作医院、国寿海棠湾健康公园、海棠国际养生社区等项目加快推进,半岭温泉健康产业组团即将对外招商。房地产业健康发展,产品结构加快调整,全市商品房销售面积146万平方米,增长45.4%,全年去库存面积53.8万平方米。着力突出农业特色,南繁、冬季瓜菜、热带花卉、热带水果等产品加快发展,各类农业标准化园区达到21家,新发展农业专业合作社40家,农产品省级著名商标达到15个。金融产业继续壮大,兴业银行三亚分行正式开业,海南银行三亚分行、浦发银行三亚分行获批筹建,全年金融业增加值57亿元,增长15.5%,增速位居各行业之首。新兴科技产业方兴未艾,积极支持中科院深海所、遥感所等机构加快发展,支持中兴通讯等企业扩大生产,三亚云港数据中心一期已开工,三亚创新创业孵化基地建成投用。

(三)抓“双修”提品质,城市面貌焕然一新。统筹开展“双修”试点、“巩卫”工作和省政府生态环境六大专项整治,取得了积极进展,全国“双修”工作现场会在我市成功召开。以治理内河水系为中心,系统推进山、海、河生态修复。加强截污纳管,完善污水管网,增设污水泵站,基本完成荔枝沟、新城污水处理厂扩建;加强排污口封堵、污染源整治、违排单位查处,基本杜绝污水直排三亚河,河流水质持续好转。入选2016年海绵城市建设试点,获得中央财政支持,实施东岸湿地公园等项目建设,加快城市道路、学校、小区等海绵化改造,国家林业局已批准设立三亚东河国家湿地公园(试点)。重点修复三亚河上游的8处受损山体,完成了抱坡岭废弃石灰岩矿山治理恢复工程。修复2.6公里海岸线,完成15公里沙生植被保护和生态恢复,在一定程度上遏制了三亚湾沙滩泥化和岸线被侵蚀现象。清理搬迁了三亚河口附近的近千艘船舶,封堵污水排海口,河口和主城区的海域环境明显优化。以打击违法建筑为关键,大力推进城市修补。去年全市拆违377万平方米,拆除户外广告牌5993块。编制《三亚市城市风貌总体规划》,确定了城市建筑色调、天际线和空间界面等管控细则,完成了解放路示范段综合环境整治、建筑外立面改造、道路功能提升和业态优化调整。建成月川生态绿道和多个生态公园,形成了“两河七园”城市中心公园带。对凤凰路、迎宾路、榆亚路进行了升级改造。沿三亚湾、三亚河以及8条主干道开展建筑亮化改造,打造了城市夜晚“金色屋顶”。以加强“巩卫”工作为重点,全面完善治理管理。坚持“全民动员、全市巩卫”,狠抓环境卫生整治、环卫设施建设、各类市场治理、重点场所监管、病媒生物防制等工作,严格执法,加强督查,电视问政,落实“巩卫”责任,取得良好成效。加快建设市政府二办停车楼、金鸡岭路供电局停车楼等项目,加强停车秩序、交通秩序管理。完成6家农贸市场升级改造,对农贸市场、水果店、餐饮服务行业、旅游购物点等进行全面整治,对道路两侧树池、电表箱、垃圾箱、公厕、围墙、公安岗亭等设施进行完善,市容市貌明显改善。

(四)抓改革促开放,发展活力不断增强。“多规合一”改革扎实推进,编制完成了《三亚市总体规划(空间类2015-2030)》,初步形成“多规合一”审批服务“一张申请表”。深入推进行政审批制度改革,积极开展区域评估评审试点,建立市、区两级权责清单。围绕“服务社会投资百日大行动”,探索实行“后置审批承诺制”。正式实施“五证合一、一照一码”商事登记制度,去年全市新增市场主体12888户,新增注册资本536亿元。积极推进三亚港务局等国企改制重组,基本完成市公交集团公司整合、公共码头资产整合。南新农场已完成整体移交,南新居正式揭牌。成立三亚城乡发展股权投资基金,引入中国PPP基金参与我市建设。全面实施“营改增”。组建市卫计委,推动公立医院医疗服务价格改革,取消了药品加成。积极参与“一带一路”对外交流合作,三亚的国际友好城市增至13个,去年新开国际(地区)正班航线15条。澜湄合作首次领导人会议、澜湄国家旅游城市(三亚)合作论坛、首届海南国际马拉松赛等活动在我市成功举办。三亚旅游警察受邀出访莫斯科,并与泰国旅游警察建立互访沟通机制。

(五)抓民生增福祉,全民共享发展成果。2016年民生支出占地方一般公共预算支出的71.8%,其中投入42.7亿元实施了十大为民办实事事项。围绕“两不愁、三保障”的总体目标,全力开展扶贫工作,在全省率先开发“扶贫数据库管理系统”,精准识别,精准帮扶,分级建立脱贫攻坚责任制,开展干部驻村帮扶,发挥企业带动作用,整合保障扶贫资金,扎实推进扶贫项目,超额完成年度脱贫计划。全市1053户4678人脱贫,8个整村推进村全部摘帽,2016年扶贫对象年人均纯收入达到7407元。加强就业社保工作,去年全市新增城镇就业岗位3.2万个,建立了高校毕业生创业孵化基地,城乡居民养老保险、基本医疗保险和大病保险基本实现全覆盖,我市已成为第二批国家级医养结合试点单位。民政救助覆盖面进一步扩大,城乡低保等标准位居全省前列。东岸棚改等项目进展顺利,圆满完成全市13514户(含农垦)棚改任务,完成农村危房改造800户,建成各类保障性住房2108套。人大附中三亚学校落户海棠湾,西南大学签约托管市第六中学。新天涯中学建设正在加快推进。崖州区、天涯区被命名为国家义务教育发展基本均衡区,吉阳区也已通过国家督导组的评估。创意产业园幼儿园等3所公办幼儿园建成开学。市人民医院被评定为三级甲等医院。国康医院主体结构已封顶。阜外医院、广安门医院已与我市达成合作意向。依托解放军总医院海南分院,建立海棠区医疗联合体。推进分级诊疗和家庭医生签约工作,加强城市医院对基层医疗卫生机构的对口支援。狠抓“菜篮子”工作,全市新增常年蔬菜基地3000亩,建立了平价蔬菜产销联盟,扩大了平价蔬菜销售网点覆盖面,蔬菜价格稳中有降。加强安全生产风险防控,狠抓社会治安综合治理。第三次荣膺“全国双拥模范城”。民族宗教、外事侨务、人口计生、海防、口岸、打击走私、防震减灾、食品药品安全、气象、人防、统计、档案、史志等工作取得新成绩,妇女、儿童、青少年、老龄、残疾人、工会、红十字事业实现新发展。

(六)抓作风强法治,政府建设扎实推进。认真执行市人大及其常委会的决议、决定,自觉接受人大、政协监督,2016年办复市人大代表建议91件、市政协委员提案124件,办复率100%。协助市人大常委会出台《三亚市山体保护条例》和《三亚市河道生态保护管理条例》。市政府颁布了《三亚市海岸带保护规定》和《三亚湾滨海公园保护规定》,制定了农贸市场、水果店、餐饮服务行业、旅游购物点等管理办法。认真开展“两学一做”学习教育,进一步转变作风,坚持深入基层、真抓实干、强化督查。抓好审计监督,加强廉政建设,严肃查处违法违纪案件。坚持抓早抓小,着力查处“微腐败”,处理了一批“四风”方面的突出问题。

各位代表,过去一年的成绩来之不易,这得益于中央精神的指导和引领,得益于国家相关部委的大力支持,得益于省委、省政府和市委的正确领导,是全市上下奋力拼搏的结果。在此,我谨代表市政府,向全市各族人民,向人大代表、政协委员,向各民主党派、工商联和社会各界人士,向中央和省驻三亚各单位,向驻市部队、武警官兵、公安政法干警,向所有关心支持三亚发展的国内外朋友,表示衷心的感谢,并致以崇高的敬意!

过去一年,三亚的巨大变化令人鼓舞,但我们也清醒地看到,我市发展还面临着不少困难和问题。特别是产业转型升级仍需加快,财政增收难度加大,城市功能、公共服务、治理管理亟待完善,教育、医疗、人才等方面仍存在短板,政府发展能力和行政效能仍有待提升。对此,我们将高度重视,认真解决。

二、2017年重点工作

2017年是党的十九大和省第七次党代会召开之年,是实施“十三五”规划的重要一年,是三亚升格地级市30周年。今年政府工作的总体要求是:全面贯彻党的十八大和十八届三中、四中、五中、六中全会、中央经济工作会议精神,深入贯彻习近平总书记系列重要讲话精神,按照“五位一体”总体布局和“四个全面”战略布局的要求,牢固树立新发展理念,落实全省经济工作会议和市第七次党代会、市委七届二次全会的部署,坚持稳中求进工作总基调,以推进供给侧结构性改革为主线,以“十大工程”“十项计划”为抓手,做好稳增长、调结构、提品质、促改革、惠民生等工作,切实提高发展质量和效益,加快建设精品城市,努力打造幸福三亚。2017年的主要预期目标是:地区生产总值增长7%以上;固定资产投资增长10%;社会消费品零售总额增长9%;地方一般公共预算收入增长8%;城镇常住居民人均可支配收入增长7.5%,农村常住居民人均可支配收入增长8.5%;居民消费价格涨幅控制在3.5%以下。为实现上述目标,要重点抓好以下工作:

(一)积极推进供给侧结构性改革,加快产业转型升级。实施产业发展计划,抓好产业园区、组团和小镇规划建设,精选优选项目,构建以大旅游为支柱、以新兴科技产业和热带特色高效农业为支撑的产业格局。

一是加快发展以旅游业为龙头的现代服务业。大力实施全域旅游发展计划,争取今年通过国家全域旅游示范区初次验收。继续推动海棠湾现代服务业产业园、凤凰岛邮轮母港产业园建设,确保亚特兰蒂斯项目10月开业,海棠湾梦幻不夜城、亚龙湾旅游文化综合体、红塘湾旅游度假区等项目加快推进。依法稳妥推进天涯海角、大小洞天、鹿回头等景区委托管理和升级改造,策划建设精品新景区,结合东岸棚改策划商业文化旅游综合体。加快推进美丽乡村建设工程,完成2016年16个美丽乡村示范村项目,新启动10个示范村建设。加强旅游铁路和海棠湾、崖州、机场等区域主要旅游通道的绿化、彩化、美化。严格执行农贸市场、水果店、餐饮服务行业、旅游购物点管理办法,清理淘汰一批,整顿规范一批,打造一批精品示范点。加强市民游客中心和旅游数据中心建设,加强旅游大数据应用,升级12345和12301服务热线平台,实现海鲜排档商家信息化系统全覆盖,抓好旅游常态化管理。推动“大三亚”旅游经济圈建设,加强互联互通、资源整合,开展宣传营销、产品开发、监管服务等方面的合作。积极促进旅游业与其他产业融合发展。继续推进恒大国际合作医院等项目,开工建设海棠湾医疗公园、三亚国际生命健康科学管理中心,加快半岭温泉健康产业组团发展,吸引优质健康养生机构,发展医疗健康产业。加快三亚国际会展中心建设,积极培育会展龙头企业,发展会展产业。引进各类金融机构,加快亚太金融小镇建设,发展金融产业。继续推进房地产业结构调整,着力发展高品质地产、经营性地产,推广精装修住宅。规范国有建设用地出让,严格控制普通商品住宅供地,控制普通度假酒店开发,鼓励发展特色度假酒店和旅游综合体。规范商品房开发、销售、中介行为和租赁市场,提高商品房交易办证效率。合理把控商品房上市节奏,加强宣传营销,继续推进房地产去库存工作。加快综合物流产业园规划建设,开工建设天涯文博园、三亚国际赛车运动旅游文化小镇,加快推进三亚格莱美音乐文化中心项目,推动旅游与商贸物流、文化体育等产业深度融合。

二是加快发展新兴科技产业。制定产业扶持政策,策划设立产业发展引导基金、孵化基金。市高新技术产业园要以互联网和信息产业为主导,制定产业准入目录,并根据新的定位对规划进行调整完善;尽快启动园区孵化楼项目,引进清华启迪、微软等众创孵化平台,建设互联网和信息产业核心区;加大招商力度,争取腾讯、百度等龙头企业入驻园区,形成品牌和集聚效应。创意产业园要加快中船重工、中船工业、航天科工等企业的项目进度,充分发挥三亚创新创业孵化基地的作用,鼓励和支持中小微科技企业发展。积极支持深海空间站等项目落地。遥感产业园争取在一季度实现供地。

三是做精做强热带特色高效农业。深入推进农业供给侧结构性改革,增加具有三亚特色的绿色优质农产品供给,狠抓农产品标准化生产,加强品牌开发和市场营销。积极打造“南繁硅谷”,建设南繁品种展示和种子交易平台,积极争取国家农业高新技术园区落户三亚。加速科技成果转化,调优冬季瓜菜、热带水果、花卉苗木等产品结构。稳步推进农村土地产权制度改革,加强财政资金引导和金融贷款支持,推动农业适度规模经营,培育新型农业经营主体。全面推进高标准农田建设,抓好高峰片区灌溉渠道和新村、水南、岭落等田洋整治项目。开展土壤环境综合治理,落实地力提升和化肥、农药减量措施,加强农产品质量安全监管。加快建设南部农副产品交易市场,积极构建农村电商体系,完善冷链保鲜物流体系。在积极开发岛外市场的同时,充分利用游客消费市场,促进农产品产销对接。结合产业小镇、美丽乡村建设,促进农旅融合,加快发展休闲农业。

(二)着力加强城市建设和治理管理,不断提升城市品质。坚持以“多规合一”为引领,统筹推进城市建设,继续开展“双修”,抓好“创文巩卫”,争当全国生态文明建设的先行区和示范市。

一是加快城市建设步伐。积极推进三亚新机场工程、凤凰机场三期工程、凤凰岛国际邮轮港二期工程,规划新机场、邮轮港的综合交通配套体系。加快编制崖州湾新区总体规划和重点片区详细规划,加快实施崖州中心渔港二期、南山港二期工程,加快南山景区升级建设,结合渔民安置区建设,打造渔港风情小镇。加快实施轨道交通工程,推动有轨电车T1线建设,力争T2线年内动工,早日启动海棠湾动车站项目。开工建设三亚客运交通枢纽综合商业配套项目,实施亚龙湾第二通道、绕城高速凤凰机场互通及连接线工程,加快三环路建设和胜利路、荔枝沟路、河西路改造,加快海榆东线海棠湾至亚龙湾段改建,积极推动三亚大桥、月明桥等项目。抓紧开展中部水厂及配套管网项目前期工作,建成青田水厂供水二期扩建工程,加快实施北部山区饮水安全工程。进一步完善城乡电网、天然气管网。落实智慧城市发展计划,积极推进三亚云港数据中心建设,围绕旅游、交通、城管、政务等重点,加强大数据应用;改造升级数字城管系统,完善社区(村)网格划分,实现人、地、事、物的精细化管理;完善政务资源共享平台,建设政务服务统一认证中心和用户中心,优化政务服务APP。

二是深入实施生态修复工程。全面梳理生态环境方面的突出问题,建立清单,加强整改。继续实施三亚东河、西河生态修复工程,开展全市内河(湖)污染源排查,实行“一河(湖)一案”和“河长制”,全面整治黑臭水体。完成红沙污水处理厂二厂和红塘湾污水处理厂建设,加快推进荔枝沟污水处理厂二厂项目,完善薄弱片区污水管网、关键节点污水泵站,加强农村地区污水治理。继续推进海绵城市建设工程,实施大隆水库向中心城区调水和海坡内河-三亚西河、高园水库-大茅水等水系连通项目,加强河道综合治理。全面推进三亚东河国家湿地公园(试点)建设,实施春光路、河东路等道路海绵化改造。积极推进三亚湾人工补沙后续工程及断面监测项目,推进亚龙湾岸滩恢复与保护工程,强化受损岸线修复和生态保护,加强崖州中心渔港的生态环境保护,落实污染物排海总量控制。加快半岭温泉及全市35个受损山体的修复工作,加快饮用水水源地等重点区域的“退果还林”。加强城市扬尘和机动车污染防治。落实低碳城市发展计划,积极建设海棠湾能源综合利用低碳示范区,逐步推广低碳节能装配式建筑,推动5个太阳能光伏试点示范项目,适度超前建设充电桩等设施,推广新能源汽车1350辆。加强重点用能、减排单位监管,支持节能降耗技术改造,禁止新增高能耗、高排放项目。

三是全面实施城市修补工程。保持违建管控力度,集中打击重点项目用地、棚改片区以及山体、农地、林地、河道、海岸带等区域内的违法建筑,完成海坡、月川拆违工作;全面开展“无违建社区(村庄)示范点”创建活动,落实网格化管理责任,完善居(村)民建房管理机制,努力实现新增违建“零增长”。加强城市规划和城市设计,强化城市色调、街道立面、建筑形态、天际线等方面的引导管控,规范绿化、亮化管理,协调城市景观风貌。继续推进公园绿地、绿道建设,实施解放一路综合环境整治、商品街大道商铺外墙立面改造工程,加快城市照明系统建设和改造。稳步推进地下综合管廊工程,加快建设海榆东线南段和胜利路地下综合管廊,科学规划设计,分步、分级建设全市管廊体系。大力实施棚户区改造工程,重点推进29个棚改项目,确保完成8302户的年度棚改任务,争取货币化安置比例不低于50%。加快东岸、月川、海坡等片区棚改,适时启动西瓜村、芒果村、临春村、南边海、阳光海岸等片区棚改。坚持政府主导、企业参与,用好贷款资金和PPP合作等模式,通过棚改完善配套设施,引进产业项目,创造就业岗位,把项目做精做优。加强棚改居民就业培训和创业引导,让群众长期生活有保障。

四是不断强化治理管理。实施好“巩卫”建设项目,落实好行业管理、属地管理、网格管理、“门前三包”四个责任,深入开展专项整治,做到常态化严格执法。全面抓好主次干道、背街小巷、城中村、城乡结合部、城市出入口沿线等部位的环境卫生管理,集中治理乱搭乱建、乱停乱放、乱扔乱吐、乱贴乱画、占道经营等现象,着力解决农贸市场设施陈旧、卫生状况差、经营秩序乱等问题,就地、就近对原有的夜市和马路市场进行改造或迁建,打造一批配套完善、管理规范的标准化便民服务点,强化食品卫生和重点场所监督管理,完善公共卫生服务体系,深入开展健康教育。严肃督查,严格考核,严厉问责,确保通过国家卫生城市复审。合理规划建设停车场、停车楼,加强交通管理,集中力量规范电动车通行秩序,对交通违章行为实行严管重罚。围绕文明交通等重点,积极开展创建全国文明城市工作。稳步推进社会治理管理体系建设计划,努力提升社区自治能力。进一步加强安全生产工作,改进信访工作。推进视频综合监控系统建设,强化社会治安综合治理,坚决打好禁毒大会战,防范和打击“两抢一盗”、暴力恐怖、严重刑事犯罪活动。推动军民深度融合发展。

(三)深入开展体制机制创新,加大改革开放力度。更好地发挥改革牵引作用,加强对外交流合作,激发城市活力与动力。

一是统筹推进重点改革。继续实施“多规合一”改革,落实空间类规划成果,完善“多规合一”审批机制,加快审批平台、电子证照库等信息化系统建设,并与省级系统实现对接。加快推进商事登记“多证合一、一照一码”,推行个体工商户营业执照和税务登记证“两证整合”。探索实行弹性出让年限以及长期租赁、先租后让、租让结合等土地供应方式。继续完善“营改增”工作,优化国地税联合办税模式和企业纳税申报服务,加强税收征管。根据产业发展需要,与金融机构合作设立产业基金,抓好三亚城乡发展股权投资基金的运作,完善与中国PPP基金等方面的合作。合理控制举债规模,有效防范政府性债务风险。大力推动全市社会信用体系建设。加快国企改制重组,规范稳妥处置资产、安置人员,组建三亚航空公司,启动国有资本投资公司、运营公司改革试点。推进南新农场公司化改革,抓好南新居建设。做好3个国营农场社会管理和公共服务职能移交工作。

二是大力实施国际化进程推进计划。制定国际化城市指标体系和评估体系。全力做好保障工作,为国家对外交往和重大外事活动服务。加强与国际友好城市的交流合作。加快南海佛学院建设。着力策划、引进国际知名会展活动和高端学术会议。进一步抓好三亚对外推介工作,与知名国际旅游商、客源市场主流媒体合作营销,大力开发国际旅游市场。今年争取开通到俄罗斯远东地区、英国曼彻斯特以及印度德里等地的6~8条国际(地区)航空航线。继续发展面向东南亚的邮轮旅游,开发海南环岛、我国东部沿海、南海主要岛屿邮轮线路。积极推动服务贸易创新发展。加强口岸基础设施建设,创新口岸通关模式,提升监管效能。

三是加快实施人才队伍建设计划。努力改善政策环境,优化人才结构,补齐人才短板。支持企业、高校、科研机构人才培养和人才引进。加强与中科院深海所和遥感所的合作,支持其在三亚招收研究生,设立博士后和院士工作站。把好公务员入口关,加强公务员在职培训,提高公务员综合素质。继续加强“候鸟”人才服务平台建设,完善“候鸟”人才信息库,做好供需衔接。

(四)坚持以人民为中心的发展思想,全力改善群众生活。加快发展民生事业,实施十大为民办实事事项,保基本、兜底线、促公平、补短板,让全市人民共享发展成果。

一是继续实施精准脱贫和城乡居民增收计划。对全市建档立卡贫困户的脱贫成效进行巩固提升,摘帽不摘政策,通过“企业+合作社+农民”的产业扶贫模式,培育贫困户的市场意识,增强发展能力,提升造血功能,确保人均纯收入稳定超过扶贫标准,吃穿不愁,义务教育、基本医疗、住房安全有保障。积极推动“大众创业、万众创新”,落实创业扶持政策,加强高校毕业生创业孵化基地的服务管理。重点做好大中专毕业生、农村转移劳动力、下岗失业人员等群体的就业服务。加强职业技能培训,积极开发公益性岗位。确保全市新增城镇就业岗位2万个,实现农村富余劳动力转移就业8000人。

二是提升公共服务质量。实施教育发展计划,完成天涯、吉阳、海棠中心幼儿园和崖州保港幼儿园建设,加快4所农场公办幼儿园建设。加快创意产业园小学建设、崖城中学改造和进士学校、林旺中学、林旺小学扩建。推动义务教育优质均衡发展,今年内省级规范学校要达到全市义务教育学校总数的20%。尽快开建人大附中三亚学校,深化与北师大和上海市长宁区的基础教育交流合作,加快与清华大学、华南师大附中、重庆南开中学、海南中学等学校的对接洽谈。支持职业教育、高等教育发展,加快推进三亚航空旅游职业学院新校区一期项目,支持海南热带海洋学院产教融合及国家大学科技园建设。实施医疗卫生发展计划,推动北大口腔医院三亚分院、阜外医院、广安门医院等项目落地建设,推动市人民医院与华西医院合作共建高端医院项目。市人民医院急诊综合楼要尽快投入使用,市妇幼保健院、市疾病预防控制中心要加快搬迁,国康医院要加快建设。积极推进基层卫生院建设,大力开展分级诊疗。继续深化公立医院改革。加强监管、整顿、清理,规范非公立医疗机构的医疗行为。优化计生服务管理,加强食品药品安全监管。积极开展全民健身运动和群众性文化活动,加快实施公共文化、体育服务设施工程,三亚大剧院、博物馆、青少年活动中心、全民健身活动中心等项目要有实质性进展。加快修缮崖城学宫、古城墙和骑楼街区,弘扬崖州民歌等特色文化,加强疍家文化保护。

三是提升民生保障水平。完善购租并举、货币化保障与保障房建设并举的住房保障制度,加快保障房项目建设,加快保障房房源分配,研究货币化保障办法,统筹解决好本地普通居民的住房问题,特别是解决好乡村教师住房问题。今年要建设乡村教师保障性住房1900套。同时,今年要完成农村危房改造400户。继续实施“菜篮子”计划,组织好常年蔬菜基地的生产与供应,扩大平价蔬菜销售网点覆盖面,增强蔬菜应急调运及冷库储备能力,加强市场价格监测预警,确保蔬菜价格稳定。实施全民参保登记计划,巩固扩大社保覆盖面,继续推进医保支付方式改革。加快实施市荔仙园公墓二期、市仙逸园管理所整体搬迁项目,继续推进殡葬改革工作。

(五)以人民群众满意为目标,加强政府自身建设。努力提升行政能力和服务水平,适应发展新形势、群众新要求。

一是深入推进依法行政。依法全面履行政府职能,落实市、区两级权责清单。健全依法决策机制,严格开展责任追究和责任倒查。继续改进综合执法体制,完善执法程序,明确操作流程,围绕“巩卫”等重点工作,强化执法取证记录,做到严格执法、规范执法、文明执法。加强行政执法人员和协警、协管员的教育、培训、整顿,提高法治意识和执法水平。落实行政执法责任制,加强执法监督和责任追究。严格执行各项地方性法规,积极推动城市管理等方面的立法工作。进一步加强普法工作,提升全民法律素质。严格执行市人大及其常委会的决议、决定,认真办理人大代表建议和政协委员提案。

二是着力提高行政效能。推进“强区扩权”,增强区级力量。实行市与区分税制财政管理体制,保障区级财力。深化行政审批制度改革,提升“放管服”水平,进一步精简和取消行政审批事项,探索“一书三清单”承诺制办理,强化事中事后监管。围绕现代服务业产业园和创意产业园,推进区域评估评审试点和园区“极简审批”改革。进一步完善政府工作督查机制,加大督查力度,特别是抓好重大决策和重点工作跟踪督办,落实各项问责制度。

三是不断改进政府作风。增强“四个意识”,坚决落实好中央、省委省政府和市委的决策部署。坚持走群众路线,践行“三严三实”,落实中央八项规定,持续整治“四风”,勇于担当,真抓实干,坚决反对懒政怠政。强化对行政权力的制约,创新审计监督方式,完善政府内部监督,自觉接受人大、政协和社会监督,完善纠错问责机制。切实加强党风廉政建设,保持反腐高压态势。

各位代表,今年是三亚升格地级市30周年,城市转型发展的新征程已经开启。让我们更加紧密地团结在以习近平同志为核心的党中央周围,在省委、省政府和市委的坚强领导下,撸起袖子加油干,为建设国际化热带滨海旅游精品城市而努力奋斗,以优异成绩迎接党的十九大和省第七次党代会胜利召开!
